# A Multi-Target Regression Method to Predict Element Concentrations in Tomato Leaves Using Hyperspectral Imaging

**DOI:** 10.34133/plantphenomics.0146

**Published:** 2024-01-29

**Authors:** Andrés Aguilar Ariza, Naoyuki Sotta, Toru Fujiwara, Wei Guo, Takehiro Kamiya

**Affiliations:** ^1^ Graduate School of Agricultural and Life Sciences, The University of Tokyo, 1-1-1, Yayoi, Bunkyo-ku, Tokyo 113-8657, Japan.; ^2^ Institute for Sustainable Agro-Ecosystem Services, Graduate School of Agricultural and Life Sciences, The University of Tokyo, 1-1-1, Midoricho, Nishitokyo-shi, Tokyo 188-0002, Japan.

## Abstract

Recent years have seen the development of novel, rapid, and inexpensive techniques for collecting plant data to monitor the nutritional status of crops. These techniques include hyperspectral imaging, which has been widely used in combination with machine learning models to predict element concentrations in plants. When there are multiple elements, the machine learning models are trained with spectral features to predict individual element concentrations; this type of single-target prediction is known as single-target regression. Although this method can achieve reliable accuracy for some elements, there are others that remain less accurate. We aimed to improve the accuracy of element concentration predictions by using a multi-target regression method that sequentially augmented the original input features (hyperspectral imaging) by chaining the predicted element concentration values. To evaluate the multi-target method, the concentrations of 17 elements in tomato leaves were predicted and compared with the single-target regression results. We trained 5 machine learning models with hyperspectral data and predicted element concentration values and found a significant improvement in the prediction accuracy for 10 elements (Mg, P, S, Mn, Fe, Co, Cu, Sr, Mo, and Cd). Furthermore, our multi-target regression method outperformed single-target predictions by increasing the coefficient of determination (*R*^2^) for elements such as Mn, Cu, Co, Fe, and Mg by 12.5%, 10.3%, 11%, 10%, and 8.4%, respectively. Hence, our multi-target method can improve the accuracy of predicting 10-element concentrations compared to single-target regression.

## Infographic

**Figure:**
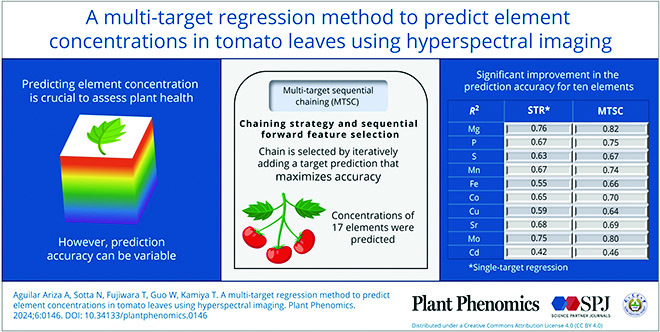

## Introduction

To ensure food security for a growing population under changing climatic conditions, strategies must be implemented to assess plant health and accurately optimize the use of resources [1,2]. Information on the nutritional status of plants is crucial for maximizing yield and ensuring plant health [3]. Currently, the measurement of nutritional status in many cases involves the chemical analysis of plant tissues to quantify element concentrations. This analysis is carried out using spectrophotometers or mass spectrometers, which destroy the plant tissue [4,5]. Although these methods provide accurate results, they are expensive and time-consuming, making large-scale measurements impractical [6,7]. To overcome this limitation, nondestructive technologies, such as hyperspectral imaging, have been adopted.

Hyperspectral imaging is a technique that captures and processes information across the electromagnetic spectrum, ranging from visible (400 to 800 nm) to near-infrared (800 to 2,500 nm) wavelengths [8]. The resulting product is an image with 2 spatial dimensions and 1 spectral dimension with a narrow-spectrum wavelength. The combination of images is shown as a 3-dimensional (3D) hyperspectral data cube [9]. Owing to the high dimensionality and correlation of the data generated by hyperspectral imaging, its analysis requires algorithms capable of handling large volumes of features [10]. Machine learning models are often used for this purpose because of their ability to deal with multicollinearity and non-parametric datasets. The combination of hyperspectral imagery and machine learning has facilitated efficient measurement of various plant physiological characteristics [4,10,11], including element concentrations.

Previous studies have achieved significant accuracy in predicting element concentrations in plants such as lettuce, orange, maize, soybean, wheat, and persimmon [11–17]. For example, Osco et al. [13] used hyperspectral data and machine learning models to predict nutrient concentrations in orange leaves and obtained high accuracies (*R*^2^ > 0.8) for some elements but low accuracies (*R*^2^ < 0.65) for others. Similar findings have been reported by Pandey et al. [16] and Acosta et al. [17].

These studies used machine learning models to predict individual element concentrations using single-target regression (STR). This method is typically employed when only one output or target is predicted. However, in scenarios with multiple targets, this regression method does not utilize other target information [18].

This limitation has been addressed by implementing multi-target regression methods that not only use common features as inputs to train learning models but also consider the statistical dependencies across targets [17,19–22]. This relationship between the targets can be represented by including the predicted target values in the input features [21]. For example, Spyromitros-Xioufis et al. [22] proposed a chaining strategy in which the original input features were incrementally augmented with predicted targets. The selection and order of targets were determined randomly. However, random selection may create chains that do not capture the correlations among the targets [23]. To avoid this randomness, Melki et al. [23] proposed an alternative approach that considers the linear correlation coefficients between targets. A linear coefficient was used to select and order the targets in the chain. However, this approach assumes linear dependencies among targets, limiting the use of machine learning models that can exploit nonlinear relationships across input features [24].

Although the effectiveness of multi-target regression methods has been demonstrated in various applications [21–23,25,26], their potential for predicting multiple element concentrations in plant tissues using hyperspectral data has not yet been explored. In this study, we predicted the element concentrations in tomato leaves from hyperspectral images using a multi-target regression method. Our method combines a chaining strategy and sequential forward feature selection, in which the chain is selected by iteratively adding a target prediction that maximizes accuracy. The concentrations of 17 elements, determined by inductively coupled plasma-mass spectrometry (ICP-MS), were predicted using the multi-target regression method and compared with the prediction accuracy of the STR. Our results indicated that the multi-target regression method significantly improved the accuracy of prediction for 10 element concentrations compared with single-target regression.

## Materials and Methods

### Tomato leaf samples

Tomato seeds were sown and grown in pots containing vermiculite supplemented with MGRL medium [27]. The pots were placed in a growth chamber at 22 °C under 16-h light and 8-h dark conditions. Thirty days after planting, leaflets were collected from each plant and used to obtain hyperspectral images, followed by elemental analysis using ICP-MS.

### Hyperspectral image acquisition

The harvested leaves were used for hyperspectral image acquisition in the range of 384 and 2,518 nm. Two hyperspectral cameras were used in this study: the visible-near-infrared camera (TUK, EBA JAPAN Co., Tokyo) captured information for 375 bands that ranged between 384 and 1,100 nm, and the second camera captured 235 bands in the short infrared spectrum (SIS-I-SH, EBA JAPAN Co., Tokyo) that encompassed the spectral region between 900 and 2,518 nm.

The sampled tomato leaflets were placed on a board in a light-cube tent equipped with halogen lamps, and pictures were captured directly above the tent using vertically fixed cameras. To compensate for the uneven light source and reflectance in the field of view, an image of 18% greyboard was captured under the same conditions and used as a reference for image analysis.

The binary files from the camera output were converted into TIF files per channel, and pixel values of each channel were normalized to those of 18% greyboard reference picture using custom R [28] scripts in the EBImage package [29]. The leaflet region was detected by binarization of a channel with high contrast, and the obtained region of interest (ROI) was manually separated into each leaflet using ImageJ [30]. In most cases, each individual leaflet was divided into 2 ROIs at the center (midrib). When a leaflet was small, it remained uncut. The median of all the pixel values within the ROI was calculated per channel and used as the input value for downstream analysis.

For spectral correction, the hyperspectral data were initially standardized, and then outliers were removed using the Smirnov–Grubb test [31], which identifies values that are statistically different from the rest.

Two frameworks were subsequently employed. One was using 2 spectral correction algorithms: orthogonal signal correction (OSC) and direct orthogonal signal correction (DOSC) algorithms [32,33].

The other was using the smoothing method, Savitzky–Golay [34], followed by a transformation step using the first derivative. In the Savitzky–Golay smoothing method, 15-nm interval and third-degree polynomial parameters were used. The resultant smoothed hyperspectral data were transformed using the first derivative. We estimated the first derivative based on a difference approximation for a finite band resolution (∆*λ*) (Eq. 1) [35].

$${\left.\frac{\mathrm{dS}}{\mathrm{d\lambda}}\right|}_{i}=\frac{S\left(\lambda_{i+1}\right)-S\left(\lambda_{i}\right)}{\lambda_{i+1}-\lambda_{i}}$$ (1)

The standardization and outlier removal processes were performed using a custom R script [28]. The 2 frameworks were implemented in Python [36].

**Fig. 1.**
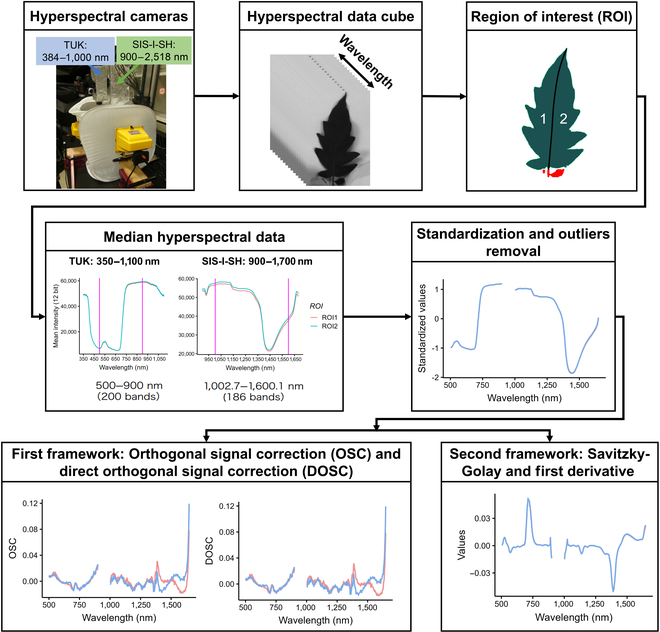
Workflow for acquisition and processing of hyperspectral data. Two cameras, TUK and SIS-I-SH, were used to acquire spectral data in the visible and near-infrared spectrums, respectively. Following hyperspectral data acquisition, the leaflet was divided into regions of interest (ROIs), and the median was calculated for each wavelength image. The data were standardized, and the outliers were removed. Two different frameworks are applied: orthogonal signal correction and the combination of signal smoothing with first derivative transformation.

Figure 1 summarizes the hyperspectral data process from image acquisition to first derivative transformation.

### Element determination by ICP-MS

After the image acquisition, the leaves were dried at 72 °C in an oven for up to 72 h, and then digested with nitric acid, followed by H_2_O_2_ at 100 °C. After complete digestion, the precipitate was dissolved with 0.08 M nitric acid, and the concentrations of 17 elements (Li, B, Na, Mg, P, S, K, Ca, Mn, Fe, Co, Cu, Zn, Rb, Sr, Mo, and Cd) were measured by ICP-MS (Agilent 7800) using 2 parts per billion indium (In) as an internal standard.

### Data split

To evaluate the accuracy of the models, we partitioned the spectral data from 742 ROIs into 2 groups: one for training (90%) and another for testing (10%). The training dataset was divided into 10-fold using a k-fold cross-validation approach.

### Multi-target regression based on a sequential chaining strategy

**Fig. 2.**
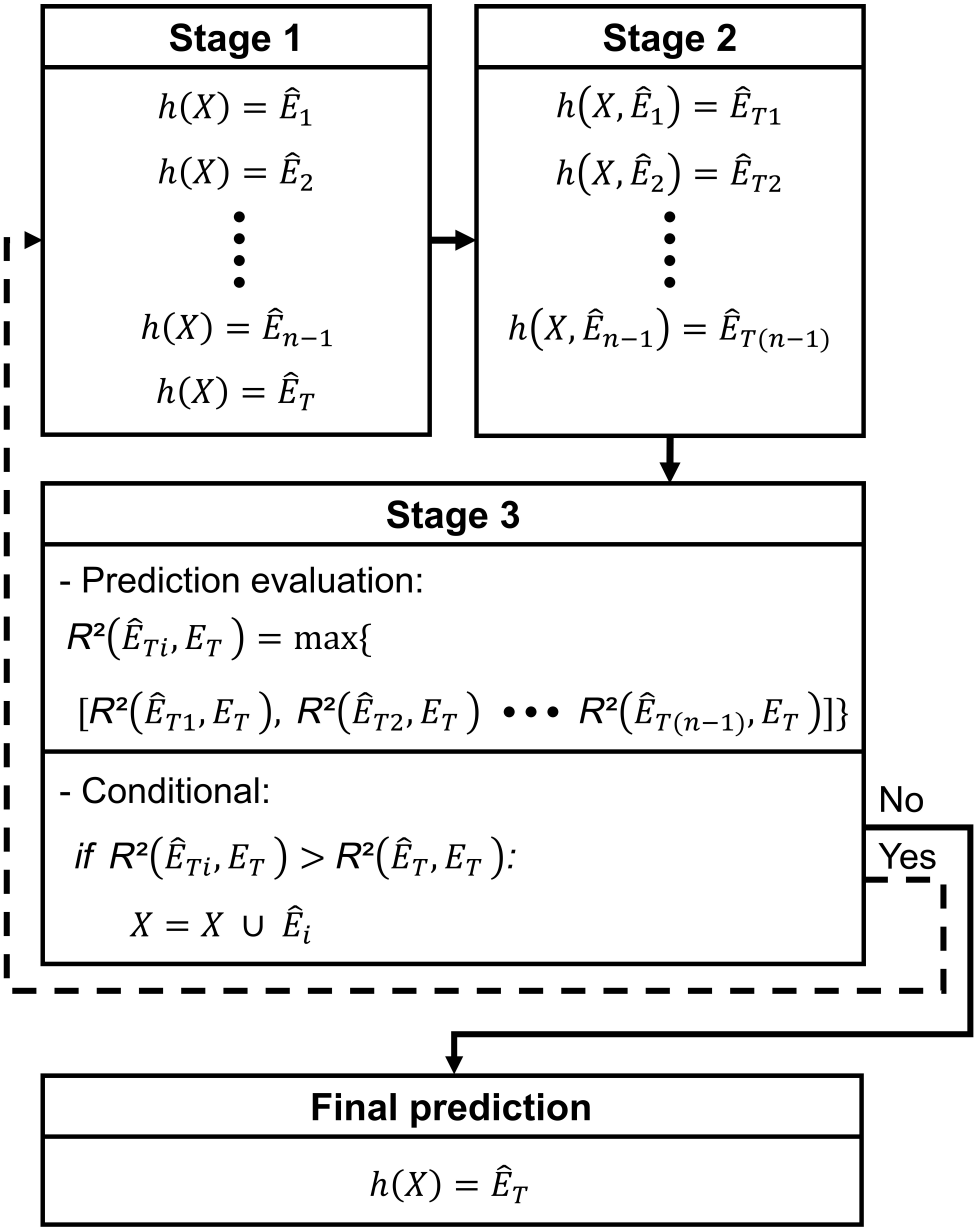
Representation of the multi-target sequential chaining (MTSC). In stage 1, the learning models (*h*) were trained with the hyperspectral data (*X*) to predict each one of the 17 (*n*) element concentrations ($\hat{E}$). In stage 2, the input features were augmented with $\hat{E}$ and then used to train the learning models to predict a target element (*E_T_*). In stage 3, the highest accuracy yielded by the new learning models trained with augmented features $\left[R^{2}\left({\hat{E}}_{\mathrm{Ti}},,,E_{T}\right)\right]$ was compared with the target element accuracy $\left[R^{2}\left({\hat{E}}_{T},,,E_{T}\right)\right]$ obtained in stage 1. If it was higher, the predicted element was permanently included as a new input feature ($X\cup {\hat{E}}_{i}$). Stages 1 to 3 were repeated until no further improvement in the prediction accuracy of *E_T_* was observed.

To include the relationship between element concentrations, a multi-target regression method that sequentially augments the original input features with predicted values was implemented. Because our method uses a sequential forward feature selection approach [37] to construct the chain, it is called multi-target sequential chaining (MTSC). The MTSC method iterates in 3 stages (2 for model training and one for filtering) until the best chain for predicting a specific target $\left({\hat{E}}_{T}\right)$ is obtained (Fig. 2). In stage 1, the learning models (*h*) were trained to predict the concentration of each of the 17 elements using hyperspectral data as input features (*X*). One element was selected as the target (*E_T_*), whereas the remaining elements (*n* − 1) were used as candidates to augment the input features. In stage 2, new learning models were trained using the remaining predicted element concentrations obtained in stage 1 $\left\{{\hat{E}}_{1},{\hat{E}}_{2},\ldots ,{\hat{E}}_{n-1}\;\right\}$ and hyperspectral data (*X*). Each new learning model $\left\{h\left(X,,,{\hat{E}}_{1}\right),,,h\left(X,,,{\hat{E}}_{2}\right)\ldots h\left(X,,,{\hat{E}}_{n-1}\right)\right\}$ yielded new predicted values for the target element $\left\{{\hat{E}}_{T1},,,{\hat{E}}_{T2},,,{\hat{E}}_{T3}\ldots {\hat{E}}_{\mathrm{Tn}-1}\right\}$. In stage 3, the predicted element (${\hat{E}}_{i}$) that is trained to predict *E_T_* is selected. Each of the $\left\{{\hat{E}}_{T1},,,{\hat{E}}_{T2},,,{\hat{E}}_{T3}\ldots {\hat{E}}_{\mathrm{Tn}-1}\right\}$ values obtained in stage 2 was evaluated by calculating the coefficient of determination (*R*^2^). The element with the highest average *R*^2^ from the 10-fold cross-validation was selected and compared with the prediction accuracy of the target element obtained in stage 1. If an improvement was observed $\left[R^{2}\left({\hat{E}}_{\mathrm{Ti}},,,E_{T}\right)>R^{2}\left({\hat{E}}_{T},,,E_{T}\right)\right]$, the input features are augmented with the element concentration prediction that maximizes the $R^{2}\left(X\cup {\hat{E}}_{i}\right)$. Stages 1 to 3 were repeated until there was no further improvement in *R*^2^. The MTSC method is implemented in Python [36].

### Learning models

Five machine learning models, partial least squares (PLS) [38], Ridge [39], Lasso, random forest (RF) [40] and support vector machine (SVM)-linear [41,42], were used as base learning models for the STR and MTSC methods. Before implementing these models, the element concentration data were scaled using min–max normalization. In addition, hyperparameter tuning was performed using an exhaustive grid search algorithm (Table 1). Scikit-learn in Python [43] was used to build learning models.

**Table 1.** The hyperparameter values used for finding the best training configuration for each learning model

Models	Tuning hyperparameters	Values	
Minimum		Maximum
Partial least square (PLS)	Number of components	1.00	20
Ridge	Regularization strength (L2)	−4	−0.5
Lasso	Regularization strength (L1)	−4	−0.5
Support vector machine-linear (SVM-linear)	Kernel coefficient (gamma)	0.0001	0.1
Regularization parameter	0.1	1,000
Random forest (RF)	Number of trees	300	
Number of features required to split	0.15	0.6
Maximum depth of the tree	2	32
Number of samples required to split in the internal node	2	8
Maximum number of samples used to train each base estimator	0.7	0.9

### Evaluation metrics

Two metrics were used to address the cross-validation and test predictions: *R*^2^ and root mean squared error (RMSE) (Eqs. 2 and 3, respectively).

$$R^{2}=1-\frac{\sum_{i=1}^{n}{\left(y_{i}-{\hat{y}}_{i}\right)}^{2}}{\sum_{i=1}^{n}{\left(y_{i}-{\bar{y}}_{i}\right)}^{2}}\;$$ (2)

$$\text{RMSE}=\sqrt{\sum_{i=1}^{n}{\left(y_{i}-{\hat{y}}_{i}\right)}^{2}}\;$$ (3)

where *n* is the number of observations, *y_i_* is the individual real value, $\bar{y}$ is the average element concentration, and ${\hat{y}}_{i}$ represents the model prediction.

To evaluate the differences between the STR and MTSC methods in each learning model, we calculated the accuracy percentage differences [∆*R*^2^ (%), ∆*RMSE* (%)] obtained by the models for each of the 10-fold cross-validation datasets.

$$\Delta R^{2}\left(\%\right)=100*\frac{R_{\text{MTSC}}^{2}-R_{\mathrm{STR}}^{2}}{R_{\mathrm{STR}}^{2}}\;$$ (4)

$$\Delta \text{RMSE}\left(\%\right)=100*\frac{\mathrm{RMS}E_{\text{MTSC}}-\mathrm{RMS}E_{\mathrm{STR}}}{\mathrm{RMS}E_{\mathrm{STR}}}\;$$ (5)

## Results

### Hyperspectral data comparison

**Fig. 3.**
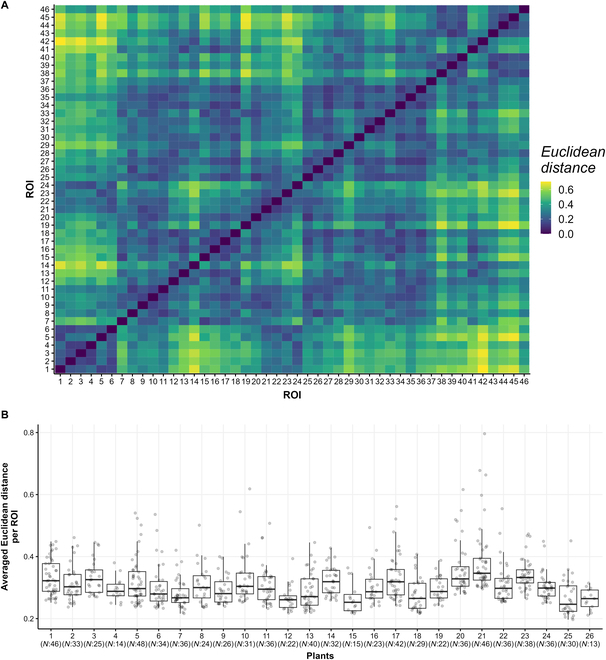
Hyperspectral data similarity comparison. (A) Example of the Euclidean distance matrix among different ROIs within a single tomato plant. (B) Comparative analysis of hyperspectral data across all 26 tomato plants used in our study. Each dot represents the averaged Euclidean distance for each ROI. *N* indicates the number of ROIs per plant.

The similarity of spectral response across leaves and plants was evaluated by comparing hyperspectral data. The Euclidean distance was used as a similarity index, with values closer to 0 indicating high similarity. Within a plant, the Euclidean distance ranged between 0 and 0.7 (Fig. 3A). Across the entire set of tomato plants (26 plants in total), we compared the average distances per ROI and plant. The Euclidean distances varied between 0.2 and 0.8 (Fig. 3B), highlighting variability in the hyperspectral data.

### Element concentration in tomato leaves

**Fig. 4.**
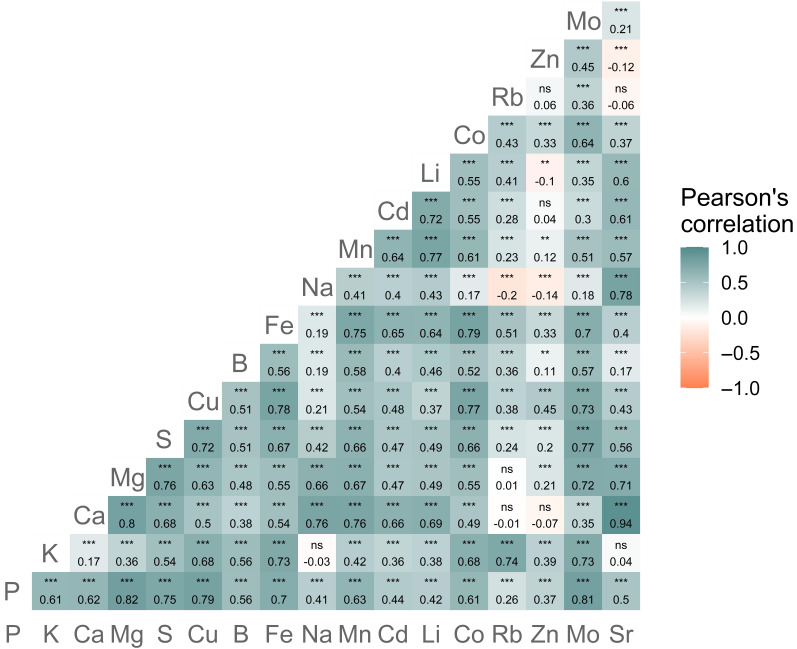
Pearson’s *R* values for concentrations of 17 elements in tomato leaflets that were determined using ICP-MS (*n* = 742 samples). We used ns to denote no significant difference between each pair correlation, and *, **, and *** to represent *P* < 0.05, *P* < 0.01, and *P* < 0.001, respectively.

The element concentrations of the 742 ROIs measured by ICP-MS showed high variability according to the coefficient of variation (Table 2). For assessing the correlation between element concentrations, the highest linear Pearson’s correlation coefficient (*R*) was observed between Sr and Ca (*R* = 0.94). In addition, Pearson’s *R* for Mg and P, Mo and P, and Mg and Ca reached values greater than 0.8 (Fig. 4). For the regression methods, the 742 ROIs were randomly split into 2 groups: 668 for training and validation and 74 for testing.

**Table 2.** Concentrations of different elements in tomato leaves

Elements	Minimum (ppm)	Mean (ppm)	Maximum (ppm)	SD (ppm)	Coefficient of variation (%)
Li	1.15	5.17	15.4	2.79	54
B	4.01	24.8	70.8	11.9	48
Na	121	481	1,510	275	57
Mg	2,320	5,560	12,200	2,090	38
P	2,610	5,910	12,800	2,010	34
S	2,350	6,530	16,300	2,600	40
K	2,680	9,820	23,700	4,110	42
Ca	6,060	19,600	50,900	8,000	41
Mn	18.3	49.9	126.3	21.2	42
Fe	43.3	97.8	239	37.9	39
Co	0.054	0.20	0.57	0.10	54
Cu	5.40	11.9	29.0	4.36	37
Zn	4.70	17.8	51.4	8.96	50
Rb	3.25	27.7	92.7	18.0	65
Sr	11.8	46.6	141	23.0	49
Mo	0.40	1.19	2.77	0.48	41
Cd	0.003	0.03	0.09	0.02	64

### Prediction of element concentrations using STR

Five learning models were used as base models for the STR: SVM-linear, PLS, Lasso, Ridge, and RF. First, to identify the optimal hyperspectral dataset for predicting element concentration, the 5 learning models were trained with 4 different inputs: standardized hyperspectral data, hyperspectral data corrected with OSC and DOSC, and hyperspectral data transformed using the first derivative. Evaluation metrics obtained from 10-fold cross-validation subsets were averaged to assess prediction accuracy. Comparative analysis of the averaged *R*^2^ results obtained by cross-validation for the 17 elements showed significant differences for the RF model when trained with the hyperspectral data transformed using first derivative (Fig. S1). No significant differences were observed in the other learning models. Based on these results, we decided to use hyperspectral data transformed using the first derivative as the input for subsequent analysis.

**Fig. 5.**
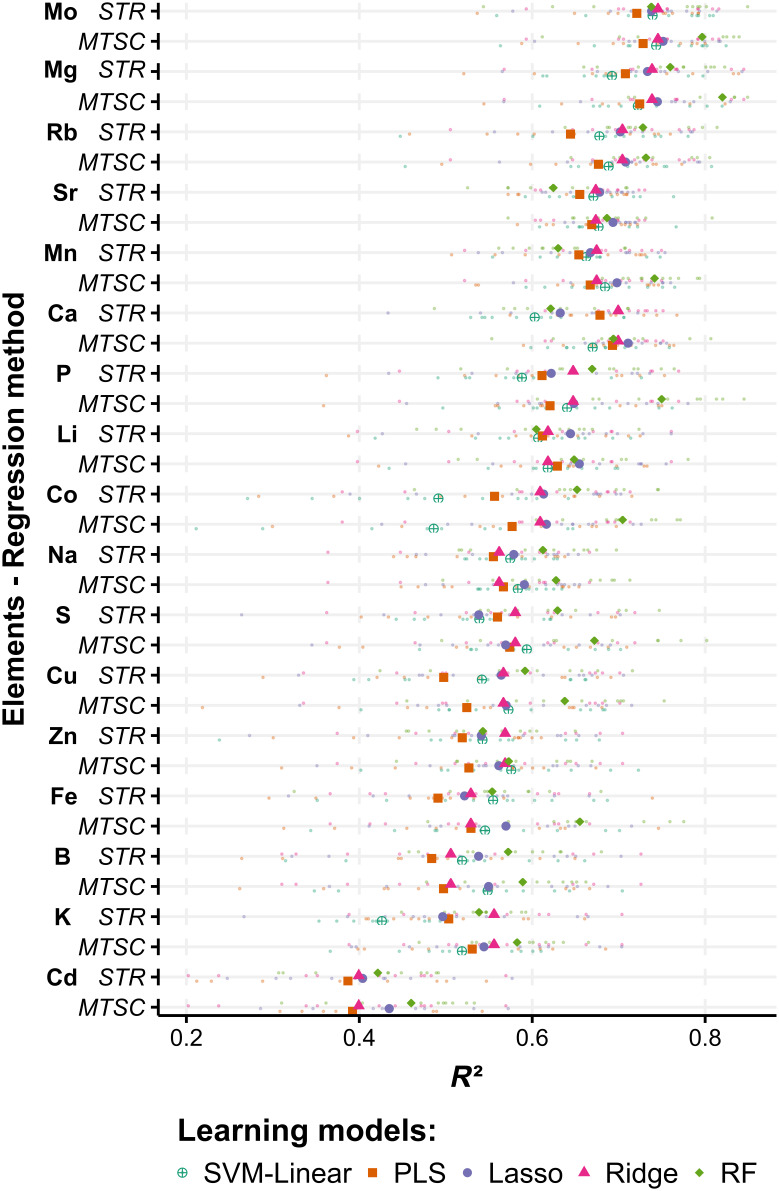
*R*^2^ of the observed and predicted element concentrations predicted by single-target regression (STR) and multi-target sequential chaining (MTSC). The *x*-axis shows the *R*^2^ of the cross-validation. Each learning model was represented by a unique color and shape. The small points represent the *R*^2^ values obtained for each of the 10-fold cross-validation subsets. The larger point represents the average of the cross-validation subsets.

The element concentrations were predicted with the 5 different models using the first derivative-transformed data. We observed that 11 elements (Mg, Mo, Rb, Ca, Sr, Mn, P, Co, Li, S, and Na) yielded predictions with *R*^2^ values ranging from 0.60 to 0.76 (Fig. 5 and Table 3), where the highest *R*^2^ (0.76) was obtained using the RF model for Mg prediction. For Cd, K, and B, the models yielded the lowest *R*^2^ values (<0.60) (Fig. 5). Compared to the other learning models, RF yielded a higher *R*^2^, whereas Ridge yielded the lowest values.

**Table 3.** Cross-validation results for single-target regression (STR) and multi-target sequential chaining (MTSC) methods. *Significant: *P* < 0.05; **Significant: *P* < 0.01.

Element	STR			MTSC				Wilcox test
Base model	*R* ^2^	RMSE	Base model	Chain	*R* ^2^	RMSE		
Li	Lasso	0.64	1.63	Lasso	Co-Mg-P	0.65	1.61	ns
B	RF	0.57	7.77	RF	S-Cu	0.59	7.61	ns
Na	RF	0.61	167	RF	Cu-Mg-P	0.63	164	ns
Mg	RF	0.76	982	RF	Cu-P	0.82	844	**
P	RF	0.67	1,125	RF	S-Rb-Cu-Mo	0.75	975	**
S	RF	0.63	1,553	RF	Co-Mg-Rb-Cu-Mo	0.67	1,441	*
K	Ridge	0.56	2,695	RF	Rb-S	0.58	2,626	ns
Ca	Ridge	0.70	4,283	Lasso	Cu-Li-Sr	0.71	4,218	ns
Mn	Ridge	0.67	11.9	RF	Na-Rb-Cu-Co	0.74	10.7	**
Fe	SVM-linear	0.55	25.0	RF	Rb-Cu	0.66	22.1	**
Co	RF	0.65	0.06	RF	Rb-Cu	0.70	0.06	**
Cu	RF	0.59	2.70	RF	Co	0.64	2.53	*
Zn	Ridge	0.57	5.77	SVM-Linear	Li-Cu-S-Ca-Rb	0.58	5.75	ns
Rb	RF	0.73	9.34	RF	S-Mg-Na-Ca	0.73	9.29	ns
Sr	Lasso	0.68	12.8	Lasso	Cu-Li	0.69	12.5	**
Mo	Ridge	0.75	0.24	RF	Rb-Cu-Co	0.80	0.21	*
Cd	RF	0.42	0.01	RF	P-Rb	0.46	0.01	**

ns, not significant. RF, random forest; SVM-linear, support vector machine using a linear kernel.

### Prediction of element concentrations using the MTSC method

To implement the MTSC method, we removed the elements whose predictions achieved low accuracy in the STR from the list of possible chain candidates. We excluded elements whose average cross-validation *R*^2^ values were less than 0.55. Therefore, Cd, K, B, Fe, and Zn were removed as possible inputs using the MTSC method.

**Fig. 6.**
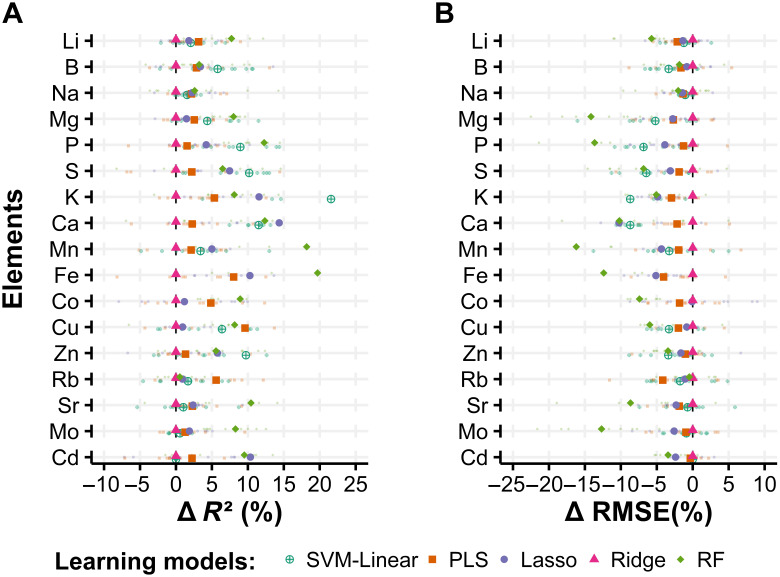
*R*² (A) and RMSE (B) differences between the single-target and multi-target k-fold cross-validation results. The percentage difference (Δ) was calculated by using cross-validation results yielded by both methods (see Materials and Methods, Eqs. 4 and 5). Each learning model is represented by a unique color and shape. The smaller points represent the *R*^2^ obtained for each of the 10-fold cross-validation subsets. The bigger point is the average of cross-validation subsets.

The MTSC results for the cross-validation dataset are shown in Fig. 5. Similarly, for the STR predictions, Mg prediction yielded the highest *R*^2^ value (0.82 for RF), whereas Cd yielded the lowest prediction *R*^2^ (0.39 PLS). To observe the improvement in prediction accuracy by the MTSC method, we compared the percentage differences between STR and MTSC (∆*R*^2^ and ∆RMSE) (Eqs. 4 and 5 in Materials and Methods). RF showed the highest improvement, averaging the 17 element results (∆*R*^2^=8.8%; ∆RMSE = −7.6%), followed by SVM-linear (∆*R*^2^=5.9%; ∆RMSE = −3.6%). In RF, elements such as Fe showed increased *R*^2^ (Fig. 6A) and decreased RMSE (Fig. 6B) by up to 19.7% and 12.4%, respectively. In contrast, the Ridge model did not show any improvement in prediction accuracy (Fig. 6). Except for Ridge, the MTSC improved the accuracy of prediction. However, the same learning model does not necessarily yield the best accuracy predictions for a specific element for both STR and MTSC. For example, in Mo, among the 5 learning models, the highest cross-validation accuracy was achieved by PLS using STR (*R*^2^ = 0.75; RMSE = 0.24), whereas for MTSC, the highest performance was achieved by RF (*R*^2^ = 0.80; RMSE = 0.21) (Table 3).

As our interest was in determining whether there was an improvement in the prediction of element concentrations using the MTSC method, we determined whether significant differences were present in the 10-fold cross-validation *R*^2^ between the STR and MTSC. We found that 10 elements (Mg, P, S, Mn, Fe, Co, Cu, Sr, Mo, and Cd) showed a significant increase in *R*^2^ when the MTSC method was implemented to predict the element concentrations (Table 3).

### Prediction comparison on the test dataset

**Fig. 7.**
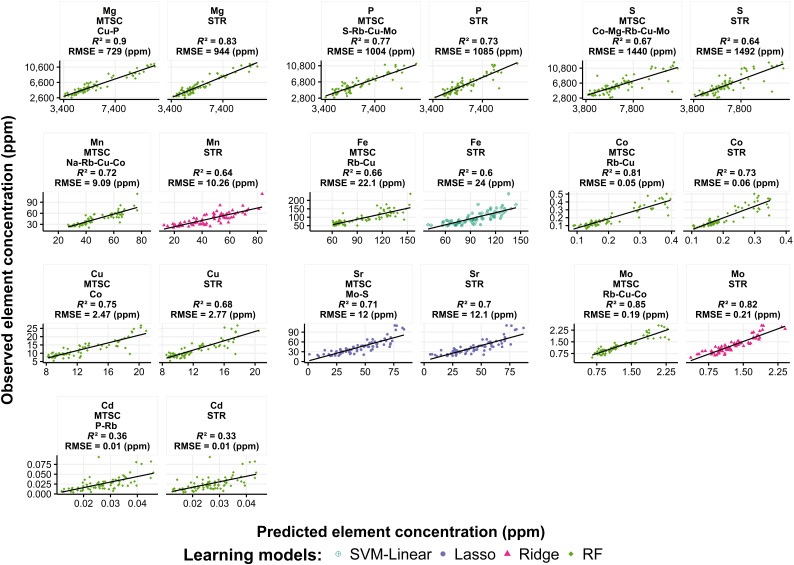
Observed and predicted element concentration values of the test dataset. The prediction was obtained using the models selected from the STR and MTSC cross-validation results. The figure shows the prediction for the 10 elements that yielded a significant difference when the STR and MTSC cross-validation results were compared.

To evaluate the predictive accuracy on previously unseen data, we predicted the element concentration values for the testing dataset (*n* = 74) using the best models obtained from STR and MTSC (Table 3). The predictions were computed for 10 elements (Mg, P, S, Mn, Fe, Co, Cu, Sr, Mo, and Cd) whose accuracy showed a significant improvement when the MTSC method was implemented. For all 10 elements, the MTSC method yielded better prediction accuracies than STR. Mn, Cu, Co, Fe, and Mg exhibited an increase in their *R*^2^ by 0.08, 0.07, 0.08, 0.06, and 0.07, respectively, and a decrease in their RMSE by 1.17, 0.3, 0.01, 1.8, and 216 parts per million (ppm), respectively (Fig. 7).

Additionally, we performed an analysis to assess the importance of features in the learning models trained with the MTSC method with Shapley additive explanation (SHAP) values [44]. We focused on the 10 elements that outperformed STR for the SHAP analysis. We found that the elements incorporated in the chain exhibited significantly higher importance than the hyperspectral features (Fig. S2), showing the effectiveness of the MTSC approach.

## Discussion

The use of hyperspectral data in combination with machine learning models is an effective method for predicting element concentrations in plants [13,15,45,46]. However, for some elements, the prediction can achieve relatively high accuracy, whereas the prediction yields poor performance for other elements. Here, the MTSC method was implemented to improve the accuracy of element concentration prediction by including the interrelationships among elements as input features [22]. By comparing the cross-validation results of the single- and multi-target regression methods for 17 elements, we observed a significant improvement in the prediction accuracy for 10 element concentrations (Mg, P, S, Mn, Fe, Co, Cu, Sr, Mo, and Cd) when MTSC was used (Table 3).

The correct selection of targets and their order in a chain play an important role in multi-target regression methods that use a chaining strategy [21]. Previous studies have addressed these challenges by randomly selecting the predicted target to be chained [21,22] or by considering only targets with strong linear correlations with the target element to be predicted [23]. In this study, the chaining strategy consisted of sequentially augmenting the input features with only the predicted targets, which improved prediction accuracy. This allowed the identification of the optimal number of targets to be included in the model. In addition, unlike selecting and ordering a chain based on a linear correlation coefficient [23], our sequential chain strategy benefits machine models that can exploit nonlinear relationships, such as RF [24,47]. For example, the best MTSC results for Fe indicated that its concentration prediction benefited from the inclusion of only 2 elements as input features: Cu and Rb (Fig. 7 and Table 3). While Fe exhibited high linear correlations with Cu, with a linear Pearson’s correlation coefficient of 0.75, its correlation with Rb was low (0.51) (Fig. 4). Regardless of the low linear correlation with Fe, the chaining of these 2 targets showed higher *R*^2^ values than those trained with a chain composed of elements (Mn, Cu, and Co) with a strong linear correlation (*R* > 0.75) (Fig. S3). This finding highlights the importance of considering nonlinear relationships in multi-target regression methods based on chaining strategies.

The extent of the improvement in accuracy achieved by the MTSC method for each element varied depending on the learning model used for the prediction (Fig. 5). The results indicate that RF achieved the highest improvement, whereas the Ridge model showed the lowest improvement (Fig. 6). These results align with previous findings that learning models based on bagged regression trees, such as RF, can better exploit the advantages of including multiple-target predictions, as reported by Spyromitros-Xioufis et al. [22]. However, models based on linear regression with a rank of feature coefficients, such as Ridge, do not effectively incorporate multi-target relationships [21,22].

Overall, our results demonstrate that the proposed MTSC method can increase the prediction accuracy over single-target regression. Though a significant increase was not observed in the prediction accuracy of all elements, none exhibited a decrease in accuracy (Fig. 7), indicating the effectiveness of the multi-target regression method for predicting element concentrations.

## Data Availability

All codes for data cleaning and analysis associated with the current submission are available at https://github.com/anaguilarar/MT_elements.
