# Supplementary material for: A Multi-Target Regression Method to Predict Element Concentrations in Tomato Leaves Using Hyperspectral Imaging
Source: Plant Phenomics. 2024 Jan 29;6:0146. doi: 10.34133/plantphenomics.0146 (PMC11020135; doi:10.34133/plantphenomics.0146)
Supplement: Supplementary 1 — Figs. S1 to S3 [file plantphenomics.0146.f1.zip › Figure_S2.pdf]

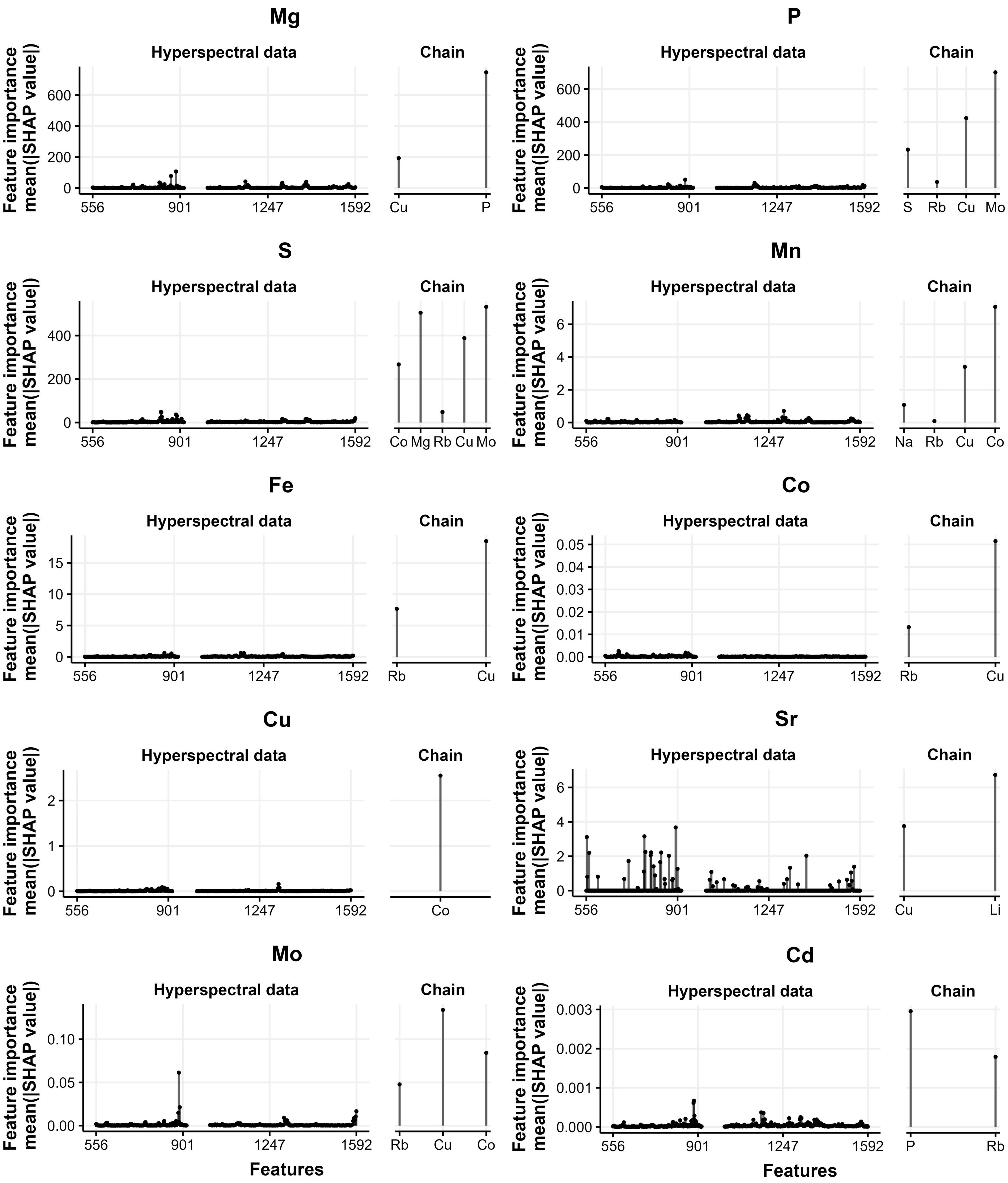
Mg
Hyperspectral data
Chain
Feature importance mean(|SHAP value|)
0
200
400
600
556
901
1247
1592
Cu
P
S
Rb
Mo
Mn
Co
Mg
Na
Fe
0.00
0.01
0.02
0.03
0.04
0.05
Cu
Sr
Li
Mo
0.05
0.10
Cd
0.000
0.001
0.002
0.003
Features
