# Supplementary figures and images for: A Multi-Target Regression Method to Predict Element Concentrations in Tomato Leaves Using Hyperspectral Imaging
Source: Plant Phenomics. 2024 Jan 29;6:0146. doi: 10.34133/plantphenomics.0146 (PMC11020135; doi:10.34133/plantphenomics.0146)

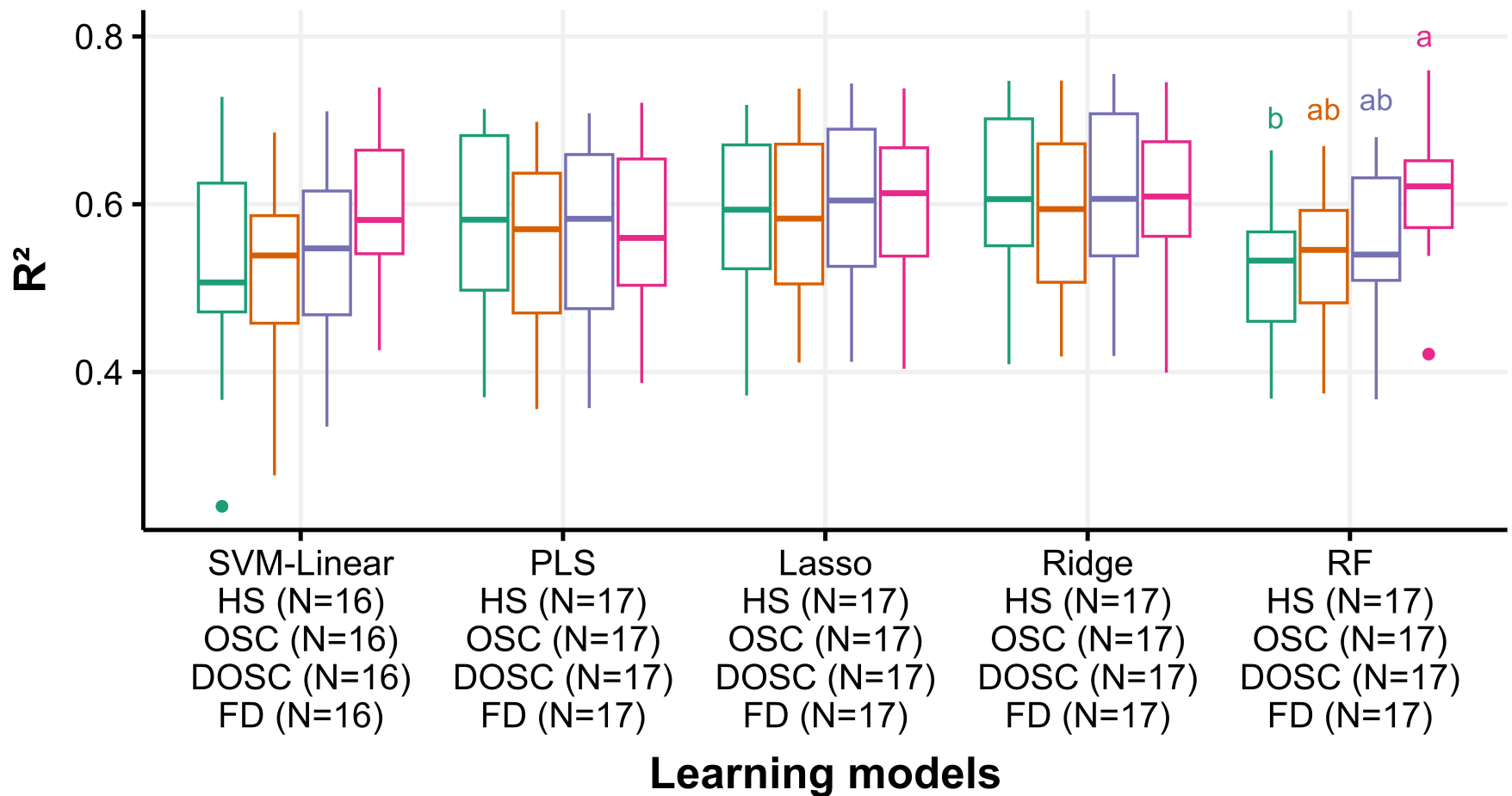

**Hyperspectral datasets:** 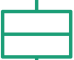 HS 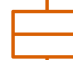 OSC 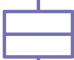 DOSC 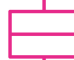 FD

Supplement: Supplementary 1 — Figs. S1 to S3 [file plantphenomics.0146.f1.zip › Figure_S1.pdf]

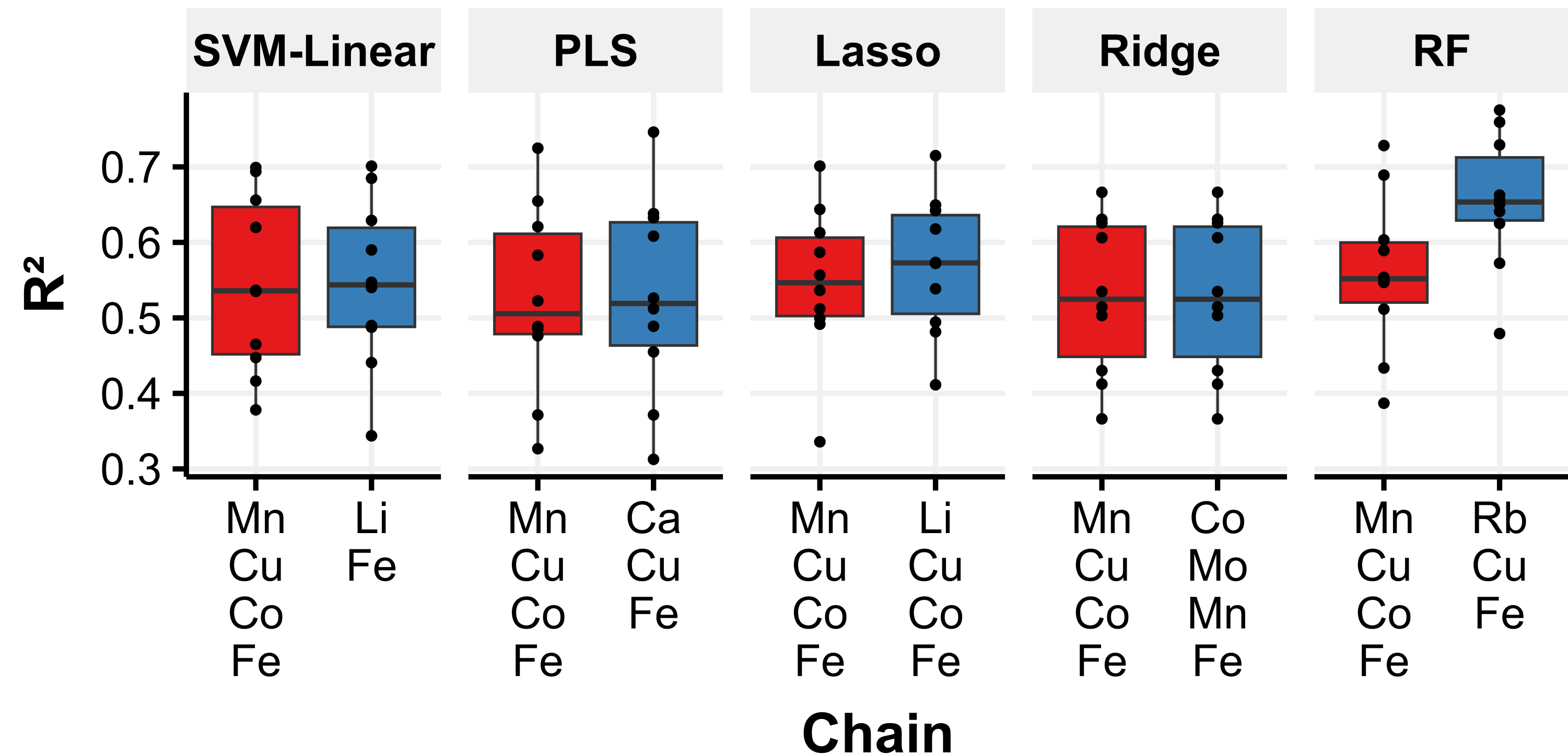

*Chaining strategy:* 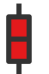 Linear correlation 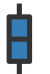 MTSC

Supplement: Supplementary 1 — Figs. S1 to S3 [file plantphenomics.0146.f1.zip › Figure_S3.pdf]
